# Interstitial Lung Diseases treatment prescription practices among pulmonologists in India: A prospective multicentre observational study (India-ILD care study)

**DOI:** 10.1371/journal.pone.0360678

**Published:** 2026-10-23

**Authors:** Vijay Hadda, Avishek Layek, Pawan Tiwari, Saurabh Mittal, Nipun Malhotra, Maroof Ahmad Khan, Prasanta Raghab Mohapatra, Sourin Bhuniya, Raj Kumar, Sonam Spalgais, Dharm Prakash Dwivedi, Vishnukanth Govindaraj, Saurabh Karmakar, Lovely Sinha, Prakhar Sharma, Girish Sindhwani, Devasahayam J. Christopher, Barney Isaac, Sheetu Singh, Bharat Bhushan Sharma, Amitabha Sengupta, Pradyot Sinhamahapatra, Nazia Mehfooz, Tehleel Altaf Shera, Vikas Marwah, Manu Chopra, Ajoy Kumar Behera, Ranganath Ganga, Puneet Saxena, Geetika Yadav, Md. Arafat Hussain, Tejas Menon Suri, Irfan Shekh, Karan Madan, Anant Mohan, on behalf of ICMR’s Network of Pulmonary Fibrosis (INPF) collaborators

**Affiliations:** 1 Department of Pulmonary Critical Care and Sleep Medicine, All India Institute of Medical Sciences, New Delhi, India; 2 Department of Bio-Statistics, All India Institute of Medical Sciences, New Delhi, India; 3 Department of Pulmonary Medicine & Critical Care, All India Institute of Medical Sciences, Bhubaneswar, Odisha, India; 4 Department of Pulmonary Critical Care and Sleep Medicine, Vallabhbhai Patel Chest Institute, New Delhi, India; 5 Department of Pulmonary Medicine, Jawaharlal Nehru Institute of Medical Education & Research, Puducherry, India; 6 Department of Pulmonary Medicine, All India Institute of Medical Sciences, Patna, India; 7 Department of Pulmonary Medicine, All India Institute of Medical Sciences, Rishikesh, India; 8 Department of Pulmonary Medicine, Christian Medical College, Vellore, India; 9 Department of Pulmonary Medicine, Rajasthan Hospital Jaipur, Jaipur, India; 10 Allergy & Pulmonary Division, Department of Medicine, Sawai Man Singh Medical College, Jaipur, India; 11 Department of Pulmonary Medicine, Institute of Postgraduate Medical Education & Research, Kolkata, India; 12 Department of Clinical Immunology and Rheumatology, Institute of Postgraduate Medical Education & Research, Kolkata, India; 13 Department of Pulmonary Medicine, Sher-e-Kashmir Institute of Medical Sciences, Soura, Srinagar, India; 14 Department of Radiodiagnosis, Sher-e-Kashmir Institute of Medical Sciences, Soura, Srinagar, India; 15 Department of Pulmonary & Critical Care Medicine, Armed Forces Medical College, Pune, India; 16 Department of Pulmonary Critical Care and Sleep Medicine, All India Institute of Medical Sciences, Raipur, India; 17 Department of Pulmonary & Critical Care Medicine, Army Hospital Research & Referral, New Delhi, India; 18 Non-Communicable Diseases, Division, Indian Council of Medical Research, New Delhi, India; Rutgers Biomedical and Health Sciences, UNITED STATES OF AMERICA

## Abstract

**Background and objectives:**

There is limited data on prescription behaviour and compliance with interstitial lung disease (ILD) guidelines among pulmonologists. This study aimed to evaluate ILDs treatment prescription behaviour in India.

**Methods:**

Prescriptions of treatment-naive ILD patients from 13 tertiary care hospitals were reviewed and classified as guideline compliant (GCP) or non-compliant (GNCP) for pharmacological and non-pharmacological components. Change in forced vital capacity (FVC), diffusing capacity (DLCO), and six-minute walk distance (6-MWD) at 6 and 12 months were compared between GCP and GNCP.

**Results:**

After screening 720 participants, 425 treatment-naïve ILD patients (56.7% female; mean age 51.7 years) were included. Connective tissue associated-ILD (CTD-ILD) was the most common diagnosis (36.2%), followed by hypersensitivity pneumonitis (17.4%), idiopathic pulmonary fibrosis (IPF, 13.9%), interstitial pneumonia with autoimmune features (13.9%), and sarcoidosis (8.2%). GCP rates for ILD specific pharmacotherapy were 83%, 74.7%, and 93.5% for IPF, CTD-ILD, and sarcoidosis, respectively. In IPF, patients with GCP had significantly lower FVC decline at 12 months compared to the GNCP (0.06 L vs. 0.25 L; p = 0.02). There was huge care-gap in non-pharmacological advice with 13.2%, 7.1%, 10.2% and 4.2% of eligible cases were advised vaccination, pulmonary rehabilitation, antigen avoidance, and lung transplantation counselling, respectively.

**Interpretation and conclusions:**

India-ILD care study revealed a huge care gap with significantly under-prescription of non-pharmacological components. These results highlight the urgent need for organized implementation strategies including dissemination of guidelines, and a greater emphasis on a structured management protocol that includes both pharmacological and non-pharmacological treatment to optimize outcomes for ILD in India.

## Introduction

Interstitial lung diseases (ILDs) constitute a heterogeneous group of diffuse parenchymal lung pathologies having more than 200 diverse aetiologies with variable disease courses and therapeutic strategies. ILDs may have a known aetiology – connective tissue diseases (CTD), hypersensitivity pneumonitis (HP), exposures (occupational, drugs, radiation etc), or idiopathic – idiopathic pulmonary fibrosis (IPF), idiopathic non-specific interstitial pneumonitis (iNSIP), alveolar macrophage syndrome, cryptogenic organizing pneumonia (COP) etc. [1]. While IPF follows a relentless progressive course, other ILDs such as CTD-ILD and HP may stabilise or partially reverse with treatment [2]. A subset of non-IPF ILDs that progresses despite treatment is termed progressive pulmonary fibrosis (PPF) [3].

Several international societies including the American Thoracic Society (ATS), European Respiratory Society (ERS), Joint Thoracic Society of Australia & New Zealand (TSANZ), American college of Rheumatology and others have published disease-specific guidelines for selected ILD subtypes – IPF, sarcoidosis, and CTD-ILD [2–5]. Guidelines recommend antifibrotic drugs (Nintedanib or Pirfenidone) for IPF and against the use of corticosteroids and immunosuppressive. For sarcoidosis, HP, CTD-ILD, and iNSIP, corticosteroids alone or in combination with another immunosuppressive agent(s) are frequently considered as the first line approach [6]. Other non-pharmacological supportive strategies suggested in guidelines for all ILD patients include pulmonary rehabilitation, vaccination, oxygen supplementation, and referral for lung-transplant, when indicated [6]. Despite guidelines, real-world evidence from developed nations consistently demonstrates significant variability in treatment practices, including under-utilization of antifibrotic therapy in IPF, inappropriate immunosuppressive use, and marked deficits in non-pharmacological care [7].

India faces unique challenges in ILD management, including heterogeneity in healthcare infrastructure, variation in drug accessibility, and a diverse epidemiological profile of ILDs vis-à-vis developed nations. Knowledge of the guideline adoption and prescription practices by physicians treating ILD patients would be pivotal for understanding the gaps. Attempt to identify the gaps in guideline awareness and application was limited by reliance on physician-reported data and did not assess their actual prescriptions in previous studies [8]. A prospective study evaluating guideline compliance using documented prescriptions and correlation of the same with prospectively measured physiological parameters was therefore planned.

This study was designed to objectively evaluate pharmacological and non-pharmacological ILD prescription-behaviour of pulmonologists across India using documented patient prescriptions, and to explore the consequences of guideline-aligned versus non-aligned treatment on the clinically important outcomes in the real-world setting.

## Materials and methods

### Study design, settings and participants

This was a prospective, multicentre, observational study conducted under the aegis of Indian Council for Medical Research’s Network of Pulmonary Fibrosis (INPF) [9] across 13 tertiary care teaching hospitals. Patient recruitment started on 26/02/2024 and ended on 24/02/2025, with final patient follow-up concluding on 31/12/2025. Treatment-naïve patients attending ILD-clinics or pulmonary medicine outpatient departments of participating centres, with an established single multidisciplinary discussion (MDD) adjudicated diagnostic label of ILD were included in the study. Patients without an established single diagnostic label or those who declined consent were excluded.

### Data collection

The copies of prescriptions from all eligible patients were used to extract the data under three domains – non-pharmacological treatment modalities, specific, and non-specific pharmacotherapy for the labelled ILD. Non-pharmacological treatment domain included – antigen avoidance advice (for HP patients in whom a causative antigen had been identified), lung transplant referral (for all patients with IPF or on home oxygen therapy), vaccination, and pulmonary rehabilitation (for all). ILD-specific pharmacological prescriptions were noted and categorised into corticosteroids, immunosuppressive agents, and antifibrotic drugs. Non ILD-specific pharmacological therapy was recorded in the following subheadings – gastric acid suppressants, *Pneumocystis jirovecii pneumoniae* (PJP) prophylaxis, calcium and vitamin D supplementation, bronchodilators, cough suppressants, and anti-pulmonary arterial hypertension specific therapies. Data was used to classify the prescriptions into guideline compliant (GCP) or non-compliant prescription (GNCP) for IPF, CTD-ILD, and sarcoidosis.

Forced vital capacity (FVC), diffusion capacity (DLCO) for carbon monoxide, and 6-minute walk test distance (MWD) were recorded at baseline, 6 months (accepted at 4–8 months), and 12 months (accepted at 10–14 months) during follow-up. Also, a telephonic follow-up was conducted at the end of the study to document non-pharmacological advice that may have been given verbally but not recorded on prescriptions.

### Definitions

For IPF, guideline compliance was based on the ATS/ERS/JRS/ALAT 2022 guidelines [3]. Accordingly, IPF patients receiving anti-fibrotic without corticosteroids and immunosuppressants were classified as GCP; whereas, omission of antifibrotic, or concurrent use of corticosteroids and/or immunosuppressants were labelled as GNCP.

For CTD-ILDs, the compliance were based on ACR and ATS 2023 guidelines [5,10]. GCP for systematic sclerosis (SSc)-ILD consisted prescription of one or more immunosuppressive agent, corticosteroids (a dose of <15 mg/day prednisolone equivalent), antifibrotic (Nintedanib) either alone or in any combination; whereas any other combination was classified as GNCP. For CTD-ILDs other than SSc-ILDs, combination of corticosteroids (any dose) with one or more immunosuppressive agent, and no upfront antifibrotic prescription constituted GCP; and any other combination were labelled as GNCP.

For sarcoidosis, based on ERS 2021 guidelines [4], GCP was defined as use of corticosteroids alone or in combination with another immunosuppressive agent, and without upfront antifibrotic. Any other combination was classified as GNCP.

For this study, we used pre-defined eligibility thresholds for non-ILD-specific pharmacotherapy such as calcium and vitamin D supplementation, PJP prophylaxis, and bronchodilators use for classification of GNCP or GCP. Non-prescription of calcium and vitamin D supplementation among patients using corticosteroids >2.5 mg/day for ≥3 months was classified as GNCP. Similarly, prescription missing PJP prophylaxis among patients using prednisolone dose of >20 mg/day for ≥1 month or concurrent steroids and immunosuppressant use were classified as GNCP. Inclusion of inhaled bronchodilators, in absence of airflow limitation (FEV1/FVC < 0.7) were also labelled as GNCP [11,12].

Compliance was also assessed for advice on antigen avoidance for HP among patients with an identified antigen, lung transplant referral, vaccination, and pulmonary rehabilitation. All IPF individuals and those prescribed home oxygen therapy were deemed eligible for lung transplant referral; hence, prescription including lung-transplant referral among these group of patients were classified as GCP and rest as GNCP. Vaccination (influenza and pneumococcal) and pulmonary rehabilitation is recommended for all patients with ILDs. Therefore, omission of advice on vaccination and pulmonary rehabilitation were labelled as GNCP [13–15].

### Study outcomes

The primary outcomes of the study were descriptive reporting of prescription behaviour for pharmacological and non-pharmacological modalities for various ILD subtypes and guideline-compliant prescription rate for IPF, CTD-ILDs, and sarcoidosis for ILD-specific pharmacotherapy. The secondary outcomes included change in FVC, DLCO, and 6-minute walk test distance (MWD) from baseline to 6 and 12 months of follow-up, compared between GCP and GNCP groups in IPF and CTD-ILD patients.

### Sample size calculation

Assuming that 50% of prescriptions given to ILD patients would be GCP, for 95% confidence interval (CI) and 5% margin of error, calculated sample size was 384 (n = 3.84 * PQ/d^2^). The number 50% was chosen as any percentage above or below this value would have yielded a lower sample size.

### Statistical analysis

Data were entered in Microsoft Excel sheet and analysed using STATA version 18. The distribution of the data was assessed using the Shapiro-Wilk test. Normally distributed continuous data were expressed as mean (standard deviation, SD) and non-normally distributed data as median with interquartile range (IQR). Categorical variables were expressed as frequencies and percentages. Categorical variables were compared using Chi-square or Fisher’s exact test. Continuous variables were compared using the independent t-test or Mann-Whitney U test as appropriate. A cut-off of <0.05 was taken as significant for p-values.

### Ethical aspect

The study protocol was approved by the Institutional Ethics Committee for post graduate research, All India Institute of Medical Sciences, New Delhi. A written informed consent was obtained from all participants prior to enrolment witnessed by two independent witnesses representing both the patient and the investigator.

## Results

Out of 720 screened prescriptions, a total of 425 treatment-naïve ILD patients’ prescriptions from 13 tertiary care teaching hospitals with an established MDD diagnosis were included in the study (Fig 1, S1 Table). A total of 19 patients and 10 patients were lost to follow up at 6-month and 12-month, respectively. The mean (SD) age of the study participants was 51.7 (13.4) years with a female predominance (56.7%). The median (IQR) symptom duration prior to enrolment was 18 (8–36) months. Majority of the participants were never-smokers and only 16.9% were current or former smokers. The most common subtype of ILDs was CTD-ILDs (36.2%), with the most common CTD being systemic sclerosis (S2 Table). There was moderate impairment of physiological parameters with a median predicted FVC of 56.5%, FEV1 of 60%, and a DLCO of 39%. A minority of participants (6.6%) showed an obstructive pattern (FEV1/FVC < 0.7) on spirometry. FVC, FEV1 were available for 392 participants while DLCO was available for 218 participants. Lung biopsy was attempted in 20.7% patients using various methods and was contributory to MDD diagnosis in 15.7% patients. The commonest computed tomography (CT) pattern observed was NSIP (33.7%), followed by UIP (31.5%), and airway centric inflammation/fibrosis consistent with hypersensitivity pneumonitis (15.5%) (S3 Table in S1 Appendix). The clinicodemographic profile of the study cohort is summarised in Table 1.

**Table 1:** Clinicodemographic profile of study cohort (N = 425). N = total sample size; n = sample size in each sub-group.

Characteristics	Value
Age, years (mean, SD)	51.7 (13.4)
Female, n (%)	241 (56.7)
Body weight, kg (mean, SD)	61.2 (13.5)
Current or former smoker, n (%)	72 (16.9)
FVC observed, L (median, IQR)	1.6 (1.2, 2.1)
FVC % predicted (median, IQR)	56.5 (44, 71)
FEV1 observed, L (median, IQR)	1.4 (1.0, 1.8)
FEV1% predicted (median, IQR)	60 (46, 75)
FVC/FEV1 Ratio (median, IQR)	84.7 (79.6, 89.2)
DLCO % predicted (median, IQR)	39 (28, 54)
6-MWD, meter (median, IQR)	327 (290, 380)
Subtypes of ILDs	
CTD-ILDs, n (%)	154 (36.2)
Hypersensitivity pneumonitis, n (%)	74 (17.4)
Idiopathic pulmonary fibrosis, n (%)	59 (13.9)
Interstitial pneumonitis with autoimmune features, n (%)	59 (13.9)
Sarcoidosis, n (%)	35 (8.2)
Idiopathic non-specific interstitial pneumonitis, n (%)	33 (7.8)
Others*	11 (2.1)

DLCO – diffusing capacity for carbon monoxide; FVC – forced vital capacity; FEV1 – forced expiratory volume in 1 second; 6-MWD – six-minute walk distance; IQR – interquartile range; *others – occupational ILDs (n = 5), cryptogenic organizing pneumonia (n = 2), post-COVID ILD (n = 2), and lymphangiomyomatosis and drug-induced ILD 1 each.

**Fig 1:**
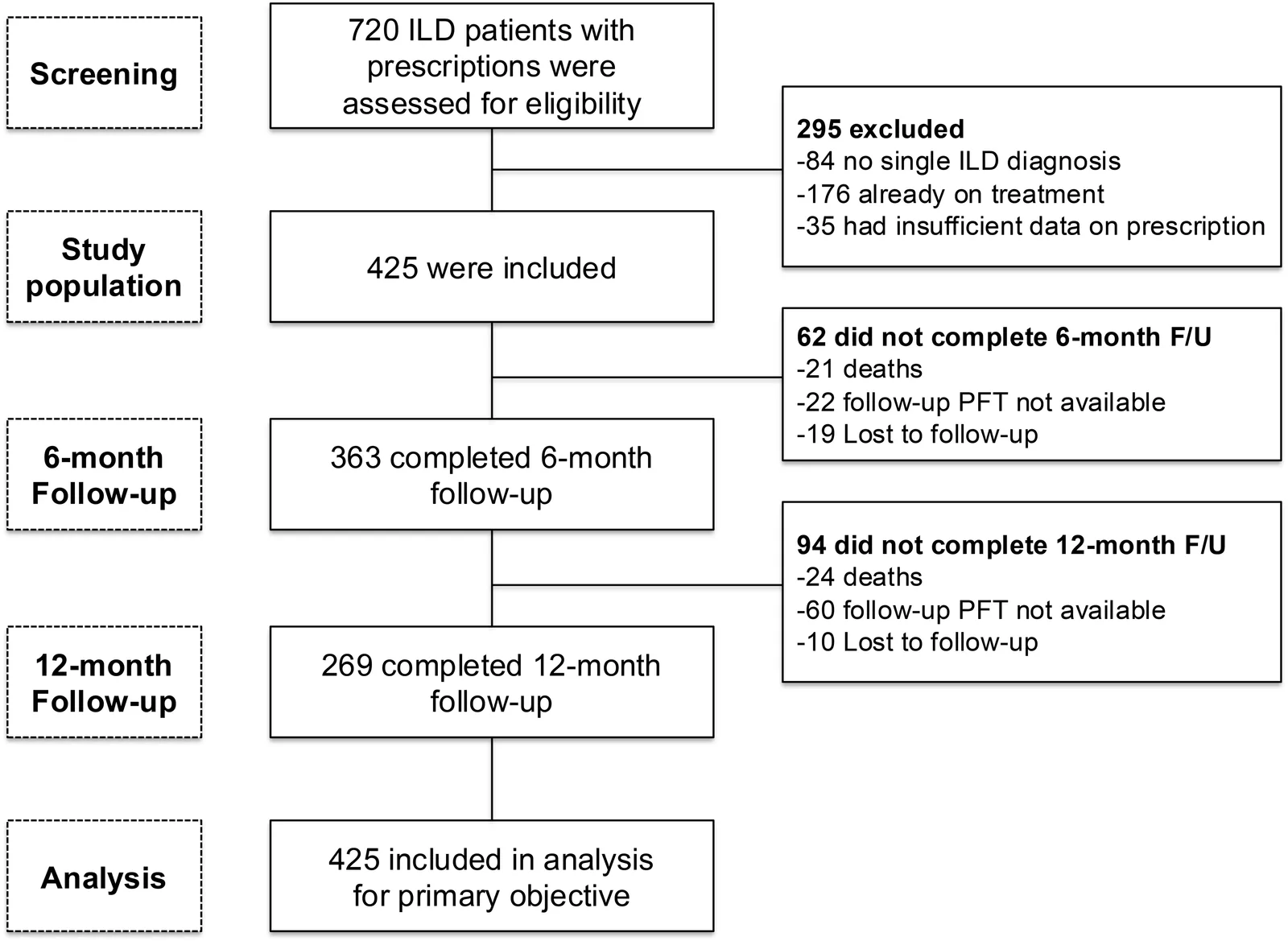
Summary of screening and enrolment of the study participants.

### ILD specific pharmacotherapy

#### Prescription practices and guideline compliance for IPF.

Among 59 IPF patients, antifibrotics were prescribed to 94.9% (n = 56) of cases. Nintedanib was more commonly prescribed (n = 48, 85.7%) as compared to pirfenidone (n = 8, 14.3%). Corticosteroids were prescribed to 7 patients (11.9%), representing GNCP. Non-corticosteroid immunosuppression was not advised to any individual. The overall GCP rate in IPF was 83.1% (Table 2).

**Table 2:** Summary of ILD specific pharmacotherapy prescription for various ILDs.

ILD specific Pharmacological treatment	IPF (N = 59)		CTD-ILD (N = 154)		Sarcoidosis (N = 31)	
	Prescribed n (%)	Median dose (IQR)	Prescribed n (%)	Median dose (IQR)	Prescribed n (%)	Median dose (IQR)
Antifibrotics	56 (94.9)	–	28 (18.2)		0	–
*-Nintedanib* (mg/day)	48	300(300, 300)	25	300 (300, 300)	–	–
*-Pirfenidone* (mg/day)	8	2100(1800, 2400)	3	1200 (600, 2400)	–	–
Steroids* -Initiation dose (mg/kg/day)	7 (11.9)	0.2 (0.1, 0.4)	113 (73.4)	0.37 (0.1, 0.6)	29 (93.5)	0.41 (0.3, 0.6)
Immunosuppressants	0	–	134 (87)	–	5 (14.7)	–
MMF (mg/day)	–	–	82	2000 (2000, 2000)	2	1000
Methotrexate (mg/week)	–	–	29	15 (15, 15)	3	15 (15, 15)
Rituximab (mg/dose)	–	–	14	1000 (1000, 1000)	–	–
Azathioprine (mg/day)	–	–	5	75 (50, 100)	–	–
Cyclophosphamide i.v (mg/dose)	–	–	2	750	–	–
Tofacitinib (mg/day)	–	–	1	10	–	–

ILD- interstitial lung diseases; IPF- idiopathic pulmonary fibrosis; CTD-ILD- *connective tissue associated-interstitial lung disease*; IQR – interquartile range; MMF-mycophenolate mofetil. *Prednisolone or equivalent.

As shown in table 3, the baseline FVC, DLCO, and 6-MWD were comparable between the two groups. The GCP group demonstrated significantly lesser FVC decline (as compared to baseline) at 6 months (median decline in FVC: 0 L vs. −0.2 L; p = 0.03) and 12 at months (−0.1 L vs. −0.3 L; p = 0.02). Six-MWD at 12 months was numerically higher in the GCP group as (median 44 m greater than GNCP) but statistically non-significant (p = 0.39). Changes in DLCO% were comparable between the two groups.

**Table 3:** Comparison of exercise capacity and physiological parameters between GCP and GNCP among IPF and CTD-ILD sub-groups.

Outcome parameters	IPF (N = 59)			CTD-ILDs (N = 154)		
	GCP (n = 49)	GNCP (n = 10)	p-value	GCP (n = 115)	GNCP (n = 39)	p-value
Baseline FVC (L)	2.2 (1.5, 2.8)	2.2 (1.9, 2.5)	0.90	1.5 (1.1, 1.8)	1.8 (1.5, 2.4)	**0.01**
FVC at 6 months (L)	2.3 (1.9, 2.9)	2.0 (1.5, 2.3)	0.12	1.5 (1.1, 1.9)	1.8 (1.6, 2.3)	**0.005**
Delta FVC at 6 months (L)*	0 (−0.1, 0.2)	−0.2 (−0.4, −0.1)	**0.03**	0.0 (−0.1, 0.1)	−0.1 (- 0.1, 0.1)	0.27
FVC at 12 months (L)	2.6 (2.1, 2.9)	1.9 (1.5, 2.0)	**0.03**	1.63 (1.3, 2.0)	1.6 (1.2, 2.0)	0.80
Delta FVC at 12 months (L) #	−0.1 (−0.2, 0.0)	−0.3 (−0.3, −0.7)	**0.02**	0.0 (−0.1, 0.2)	−0.1 (−0.3, 0.2)	0.32
Baseline DLCO (%)	37.5 (33, 56)	34 (28, 37)	0.16	39 (28, 47)	39 (29, 72)	0.28
DLCO (%) at 6 months	40.5 (30, 51)	32 (26, 33)	0.12	34 (23.4, 46)	47.9 (28, 50.5)	0.22
Delta DLCO at 6 months (%) #	−1 (−3, 0)	−3.5 (- 4.5, −2)	0.21	0.50 (−15, 7)	−2 (−11, 0)	0.32
DLCO (%) at 12 months	29 (25, 44)	36 (34, 40)	0.41	35 (24, 49)	52.5 (33, 68)	0.055
Delta DLCO at 12 months (%) #	−7 (−12, 2)	2 (−1, 3)	0.29	−1 (−5.5, 14.5)	6 (0, 28)	0.14
Baseline 6-MWD, m	345.5 (242.5, 388.5)	320 (260, 370)	0.58	315.5 (280, 367.5)	320(270, 380)	0.70
6-MWD at 6 months, m	357 (310, 378)	340 (320, 380)	0.85	320 (290, 360)	315(280, 360)	0.85
Delta 6-MWD at 6 months*, m	0 (41.7, −22.5)	4 (−10,80)	0.53	0 (−20, 42.5)	0(−20, 17)	0.49
6-MWD at 12 months, m	354 (296.5, 417.5)	330 (260, 409)	0.52	322 (305, 370)	314(240, 365)	0.29
Delta 6-MWD at 12 months #, m	44 (90.5, −42.5)	0 (−14, 55)	0.39	20 (−27, 54)	15 (−27.5, 54.5)	0.91

*Delta at 6 month – change in outcome parameter at 6 month w.r.t baseline; #Delta at 12 month – change in outcome parameter at 12 month w.r.t baseline; GCP – guideline compliant prescription; GNCP – guideline non-compliant prescription; FVC – forced vital capacity; DLCO: diffusion capacity for carbon monoxide; 6-MWD – six-minute walk distance.

### Prescription practices and guideline compliance for CTD-ILDs

Among 154 CTD-ILDs patients, corticosteroids were prescribed to 113 (73.4%) with a median initiation dose of 0.37 mg/kg/day (0.1, 0.6) of prednisolone or equivalent and immunosuppressive agents to 134 patients (87.0%) participants. Among immunosuppressive agents, mycophenolate mofetil (MMF) was the most commonly prescribed agent (n = 82, 61.2%) followed by methotrexate (n = 29, 21.6%) and rituximab (n = 14. 10.4%). Upfront antifibrotic therapy was prescribed to 28 (18.2%) patients (nintedanib: 25, pirfenidone: 3). Overall GCP rate for CTD-ILDs was 74.7% (Table 2).

As shown in table 3, among CTD-ILDs, the baseline FVC was significantly lower in the GCP group (1.5 L) compared with the GNCP group (1.8 L, p = 0.01). At 6 months, the median FVC remained lower in the GCP group than in the GNCP group. However, at 12 months, median FVC values were comparable between the two groups. The change in FVC from baseline to 6 months as well as baseline to 12 months was not significantly different between the GCP and GNCP groups. At 12 months, the median FVC in compliant group improved by 30 ml whereas in the non-compliant group it declined by 70 ml showing a trend towards betterment in GCP group. (absolute difference 100 ml, p = 0.32). Changes in DLCO% and 6-MWD did not significantly differ between the two groups.

### Prescription practices and guideline compliance for Sarcoidosis

In Scadding stage 2/3 Sarcoidosis (n = 31), corticosteroids were prescribed to 93.5% (n = 29) of patients with a median (IQR) initiation dose of 0.41 (0.29, 0.55) mg/kg/day of prednisolone or equivalent. Up-front immunosuppressants (methotrexate or MMF) were prescribed to 5 (14.7%) patients. There was no prescription of antifibrotic. Overall, GCP rate observed was 93.5%.

### Prescription practices for other ILDs

#### ILD specific pharmacotherapy.

Among patients with HP (n = 74), corticosteroids were prescribed to 91.9% (n = 68) with a median dose of 0.47 mg/kg/day (0.4–0.5) of prednisolone or equivalent. MMF was the sole immunosuppressant drug, prescribed to 35 (47.3%) whereas antifibrotic was prescribed to 8 (10.8%) participants. The most common regimen consisted steroid alone (41.9%, n = 31), followed by steroid plus immunosuppressant (39.2%, n = 29).

Among 92 patients with iPAF or iNSIP (we adopted combined reporting due to absence of specific guidelines), corticosteroids were prescribed to 78 (84.8%) participants, with a dose of 0.5 mg/kg/day (0.3–0.5) of prednisolone or equivalent. Immunosuppressive agents were prescribed to 69 (75.0%) participants with MMF leading the chart (62 of 69, 89.9%). Upfront antifibrotic was prescribed to 12 (13.0%) participants. The most common regimen was corticosteroids plus immunosuppressant (59.8%, n = 55), followed by steroids alone (17.4%, n = 16).

Across all ILD diagnoses (CTD-ILD, HP, iPAF and iNSIP), the median corticosteroids initiation dose did not significantly differ (p = 0.056); however, the minimum maintenance dose was significantly lower in CTD-ILDs (5 mg/day) as compared to HP, iPAF, and iNSIP (10 mg/day; p = 0.0001). Also, the time to taper from initiation dose to minimum maintenance dose was significantly shorter in CTD-ILD (6 weeks) compared to other diagnoses (p = 0.0001).

Univariate analysis conducted in IPF and CTD-ILD combined subgroup (n = 213) found no significant association between guideline compliance and sex, smoking status, symptom duration, CT pattern (UIP or NSIP), or baseline physiological parameters (FVC, DLCO, 6-MWD).

### Non-ILD specific pharmacological therapies

There were 263 participants who were eligible for PJP prophylaxis prescription. However, only 69 (26.2%) were prescribed PJP prophylaxis. Calcium and vitamin D supplementation was prescribed to 262 (85.1%) out of 308 eligible patients. Almost three-fourth (72%) of potentially eligible participants were prescribed gastric acid suppressants, predominantly proton pump inhibitors 298/306 (72.0%). Cough suppressants were prescribed to 83(19.5%) of participants. Pulmonary arterial hypertension-specific therapy was prescribed to 21 patients (4.9%), of which most were SSc-associated CTD-ILD (20/21). A total of 27 (6.4%) were prescribed multivitamins (S4 Table in S1 Appendi).

### Non-pharmacological treatment prescription behaviour

As shown in table 4, non-pharmacological interventions were markedly under-prescribed. Vaccination, pulmonary rehabilitation, and antigen avoidance advice were recorded in 13.2%, 7.1%, and 10.2%, respectively for eligible participants. Lung transplant referral was recorded in only 3 of 71 eligible patients (4.2%).

**Table 4:** Summary of non-pharmacological treatment prescriptions.

Modality	Eligible (N)	Prescribed, n (%)
Any vaccination advice	425	56 (13.2)
*-Pneumococcal vaccine*	425	47 (11.1)
*-Influenza vaccine*	425	50 (11.8)
Pulmonary Rehabilitation	425	30 (7.1)
Antigen avoidance advice^*^	49	5 (10.2)
Transplant referrals^#^	71	3 (4.2)

* *Patients with diagnosis of hypersensitivity pneumonitis with known antigen exposure, #Patients with IPF or who were advised oxygen*

Home oxygen therapy was prescribed to 12 patients. The complete documentation of all four components (interface, duration, target SpO2, flow rate) was present in only 50% of these prescriptions. Home NIV was prescribed to 4 patients but complete documentation of all components (NIV mode, settings, interface and use of humidifier) was noted in only 1 prescription. Notably, there was no prescription for ambulatory oxygen therapy despite 41.7% showing significant exertional desaturation (>5% SpO2 fall during 6MWT).

Telephonic follow-up at the end of the 12 months study period revealed substantially higher patient-reported rates of non-pharmacological treatment advice. During telephonic call, 40.6%, 51.5%, and 64% recalled vaccination, some form of pulmonary rehabilitation, and avoidance of potential antigens advice, respectively. Lung transplant referral rates remained consistently low even on telephonic follow-up (7.7% of eligible patients) (S5 Table in S1 Appendi).

## Discussion

India-ILD care study is the first multicentric, prospective data-set assessing ILD treatment prescription practices and guideline compliance rates among physicians in India based on real time prescription. Our results show that the ILD-specific drug prescriptions were in line with existing guidelines. However, for the non-pharmacological strategies and non-ILD specific therapy, the majority of prescriptions are GNCP.

The pharmacological therapy has been most extensively studied for IPF and multiple guidelines have recommended anti-fibrotics, either pirfenidone or nintedanib, as the first line therapy [3]. PANTHER trial has shown that corticosteroids were associated with adverse outcomes including increased risk of mortality [16]. Despite these, corticosteroids in more than 10% of prescriptions is concerning. On the other hand, our results showed that 83% of IPF prescriptions were guideline compliant for IPF-specific pharmacotherapy. Previously reported rates from physician surveys and registry-based data have shown a compliance rates as low as 20–63% [7,17,18]. The higher GCP rate observed in our study likely reflects the predominantly tertiary care setting, where awareness of contemporary guidelines may be greater. Our study also reported nintedanib as a preferred antifibrotic prescribed to more than 85%, possibly reflecting greater dosing convenience compared to pirfenidone. The finding that GCP for IPF was associated with significantly lower FVC decline at both 6 months (difference: 160 ml; p = 0.03) and 12 months (difference: 190 ml; p = 0.02) is clinically meaningful. The magnitude of FVC decline in the non-compliant group (250 ml at 12 months) is comparable to the annual FVC decline of 223.5 ml reported in the placebo arms of the pooled INPULSIS-1 and INPULSIS-2 trials, highlighting the importance of guideline-based prescription [19].

CTD-ILD prescription compliance rate (74.7%) was lower than for IPF and sarcoidosis, which may partly reflect the relatively recent publication of formal CTD-ILD guidelines and the conditional nature of many recommendations with low-quality evidence [5]. The absence of a statistically significant difference in FVC decline between the CTD-ILD GCP and GNCP groups contrasts with IPF, and may be explained by the biological heterogeneity of CTD-ILD subtypes and the relatively short 12-month follow-up for this typically more slowly progressive group vis-à-vis IPF which has a relentlessly progressive disease course. Sarcoidosis had the highest compliance rate (93.5%), consistent with the long-standing and straightforward nature of steroid-first treatment guidelines for this condition — consistent with data from prior studies [20].

The most striking finding of this study was the under-prescription of non-pharmacological modalities. Vaccination advice was recorded in only 13%, pulmonary rehabilitation in 7%, antigen avoidance (HP) in 10%, and lung transplant referral in 4% of eligible patients. Pulmonary rehabilitation is individually tailored, multimodal package that consists of assessment, endurance and strengthening exercises, and patient education. Despite challenges, data suggest that pulmonary rehabilitation increase exercise capacity, reduces dyspnea and improve quality of life among patients with ILDs; hence, there has been a strong recommendation for pulmonary rehabilitation for ILDs [15]. The GCP rates are substantially lower than reported from developed countries, and highlight systemic gaps in comprehensive ILD care [21]. However, even in developed countries the access to pulmonary rehabilitation is limited to less than half of outdoor patients [22]. The access to pulmonary rehabilitation may be worse in low and middle- income countries, similar to India, and may be a plausible reason for lesser prescription. The reported vaccination rate among ILD patients is approximately 40% for influenza vaccine [23]. Also, there seems to be behavioural issues as highlighted by observation that telephonic follow-up revealed substantially higher rate, approximately 40–64% for vaccination, pulmonary rehabilitation, and antigen avoidance; pointing to a documentation gap rather than an absolute absence of prescription. Lung transplant referrals remained low at 7.7% even on telephonic follow-up, indicating underutilisation. The reasons may include the limited facility of lung transplant among participating centres, as only one among all participating centres has an active lung transplant programme (S5 Table in S1 Appendi).

Severe exertional hypoxemia, in absence of resting hypoxemia, is common and may be responsible exercise intolerance, poor quality of life, and reduced survival among ILD patients [24,25]. The existing guidelines have contradictory recommendations for ambulatory oxygen prescription in patients with chronic lung disease [26–28]. This uncertainty might be a reason that none of the study participants was prescribed ambulatory oxygen, despite 41.7% of those undergoing 6MWT meeting the eligibility criterion of >5% desaturation. Patients with exertional desaturation with an oxygen saturation of 88% or less might be benefitted with ambulatory oxygen, especially improvement in exercise capacity and quality of life. Our study reports the care gap that may be targeted for improvement.

India-ILD care study is the first multicentric, prospective, prescription-based data from Indian subcontinent that used pre-defined compliance criteria based on contemporary guidelines making it relevant. It also incorporated telephonic follow-up to capture real world gap between prescription and informal advice practices. However, we recognize several limitations of the study. First, the study only focussed on prescription behaviour, not the patient’s compliance to the prescription. Patient compliance to the pharmacological and non-pharmacological advices is crucial for actual effects on clinical outcomes. Second, with the observational nature of study, it is difficult to establish a causal relationship. Third, all participating centres were tertiary care teaching hospital and results may not be applicable for other settings. Fourth, there are missing physiological data for some of the patients that limits the analysis of association of GCP and GNCP with these parameters. We used telephonic follow-up for assessing some of the parameters (vaccination, pulmonary rehabilitation, lung transplant referrals and antigen avoidance); however, there is potential recall bias associated with such approach. Also, since our study was a prospective observational study and not a randomized controlled trial, our findings should be interpreted as demonstrating associations rather than causality.

## Conclusions

India-ILD care study reports that guideline adherence for ILD-specific pharmacotherapy prescription was reasonably satisfactory; however, non-pharmacological care was markedly under-documented and likely under-delivered. These findings highlight the gaps in ILD care in India. There is need for improved guideline dissemination, structured implementation strategies, and a greater emphasis on prescription of comprehensive plan, extending beyond pharmacotherapy, to optimise outcomes for ILD patients in India.

## Supporting information

S1 Appendix Supplementary Appendix 1. (PDF)

S2 Appendix Supplementary Appendix 2. (PDF)
