# Supplementary material for: Interstitial Lung Diseases treatment prescription practices among pulmonologists in India: A prospective multicentre observational study (India-ILD care study)
Source: PLoS One. 2026 Oct 23;21(10):e0360678. doi: 10.1371/journal.pone.0360678 (PMC13693893; doi:10.1371/journal.pone.0360678)
Supplement: Supplementary file 1 [file pone.0360678.s001.pdf]

SUPPLEMENTARY APPENDIX 1

Title: Interstitial Lung Diseases Treatment Prescription Practices among Pulmonologists in India: A Prospective Multicentre Observational Study (India-ILD Care study)

A total of 425 treatment-naïve ILD patients with an established MDD diagnosis from 13 tertiary care teaching hospitals were included in the study. All India Institute of Medical Sciences (AIIMS), New Delhi contributed the largest proportion of cases (145 patients; 34.1%), followed by AIIMS, Bhubaneswar (8.5%), Vallabhbhai Patel Chest Institute (VPCI), Delhi (7.5%), and Jawaharlal Institute of Postgraduate Medical Education and Research (JIPMER), Puducherry (7.3%). The remaining patients were enrolled across multiple tertiary centres nationwide, each contributing between 0.7% and 6.8% of the total study population as detailed in supplementary S1 Table.

S1 Table: Centre-wise distribution of study cohort (N=425); n= sample size in each centre

Centre Name	n (%)
AIIMS, DELHI	145 (34.1)
AIIMS, Bhubaneswar	36 (8.5)
VPCI, Delhi	32 (7.5)
JIPMER, Pondicherry	31 (7.3)
AIIMS, Patna	29 (6.8)
AIIMS, Rishikesh	29 (6.8)
Christian Medical College, (CMC)Vellore	26 (6.1)
Sawai Man Singh (SMS) Medical College, Jaipur	24 (5.7)
Institute of Postgraduate Medical Education and Research (IPGMER), Kolkata	23 (5.4)
Sher-e-Kashmir institute of medical Sciences (SKIMS), Srinagar	20 (4.7)
Armed Forces medical college (AFMC), Pune	14 (3.3)
AIIMS, Raipur	13 (3.1)
Army Hospital Research & Referral (AHR&R), Delhi	3 (0.71)

Connective tissue disease–associated ILD (CTD-ILD) was the most common diagnosis, accounting for 36.2% patients. Within the largest subgroup i.e. CTD-ILD group (n=154), systemic sclerosis (SSc) was the most prevalent CTD diagnosis (35.7%), followed by rheumatoid arthritis (RA- 29.9%) and Myositis-associated ILD (13.4%). As shown in

supplementary S2 table, the other individual connective tissue diseases accounted for a less than 10% of cases each.

S2 Table: CTD diagnoses of CTD-ILD subgroup, N=154, n=sample size in each sub-group

**CTD Diagnoses of CTD-ILD cases**	**n (%)**
SSc	56 (35.7)
RA	47 (29.9)
Myositis	21 (13.4)
MCTD	9 (5.7)
Sjogren	7 (4.5)
UCTD	6 (3.8)
SLE	3 (1.9)
Myositis/RA overlap	3 (1.9)
Myositis/SSc overlap	1 (0.6)
Myositis/SLE overlap	1 (0.6)

Among various high resolution computed tomography (HRCT) patterns, non-specific interstitial pneumonia (NSIP) was the most common (33.7%) followed by the usual interstitial pneumonia (UIP- 31.5%). Pattern consistent with hypersensitivity pneumonitis (HP) and sarcoidosis was observed in 66 (15.5%) and 35 (8.2%) patients respectively. As outlined in supplementary S3 table, less frequent patterns included NSIP with organising pneumonia (OP) overlap, organizing pneumonia alone, lymphoid interstitial pneumonia (LIP) and cystic ILD. Approximately 2.5% of CT pattens were unclassifiable.

S3 Table: HRCT pattern of ILD patients in study population. N=total sample size, n=sample size in each HRCT pattern

**HRCT pattern**	**n (%)**
NSIP	143 (33.7)
UIP	134 (31.5)
Consistent with HP	66 (15.5)
Consistent with Sarcoidosis	35 (8.2)
NSIP and OP overlap	28 (6.6)
Unclassifiable	11 (2.6)
OP	5 (1.2)
LIP	2 (0.5)
Cystic ILD	1 (0.2)

The various non ILD specific pharmacological therapies prescription behaviour is summarised in the following S4 table.

S4 Table: Non ILD specific pharmacological therapy treatment practices among study cohort

**Pharmacological modality (total population=425)**	**Number of eligible participants (N)**	**Prescribed, n (%)**
PJP prophylaxis[$]	263	69 (26.2)
Calcium & Vitamin D supplementation*	308	262 (85.1)
Acid Suppressants<br>PPi<br>H2 blocker	-	306 (72.0)<br>298<br>8
Cough Suppressants<br>Cough syrup<br>Other oral agent	-	83 (19.5)<br>67<br>16
Anti-Pulmonary hypertension specific therapy<br>Single agent therapy<br>Dual agent therapy	-	21 (4.9)<br>18<br>3
Multivitamin supplements		27 (6.4)

According to telephonically collected end of study patient-reported data, 93 out of 231 patients (40.3%) had gotten some vaccination advice, and 119 patients (51.5%) had received advice to do some form of breathing exercises. Antigen avoidance advice was reported by 16 patients (64.0%) out of 25 patients with hypersensitivity pneumonitis and known antigen exposure who responded to telephonic follow-up. Two patients (7.7%) out of the 26 patients who were eligible for lung transplant referral and answered follow-up calls said they had had been told about lung transplant as a treatment option. The same is summarised in following S5 table.

S5 Table: End of study telephonic follow-up data: Non pharmacological treatment modalities

Modality	Eligible amongst answered calls (N)	Prescribed as per follow-up call data, n (%)	Eligible in entire cohort (N)	Prescribed as per prescription, n (%)
Any Vaccination advice	231	93 (40.6)	425	56 (13.1)
Pulmonary Rehabilitation	231	119 (51.5)	425	30 (7.0)
Antigen avoidance advice for HP$	25	16 (64.0)	49	5 (10.2)
Transplant referrals*	26	2 (7.7)	71	3 (4.2)
$diagnosis of HP with known antigen exposure<br>*IPF or advised Oxygen				

Guideline-compliant ILD-specific pharmacotherapy was prescribed in 49 (83.1%) patients with IPF and 144 (77.8%) patients with non-IPF ILDs. Among CTD-ILD and Sarcoidosis patients, guidelines compliant medications were given to 115 (74.7%) and 29 (93.6%) respectively. As shown in S6 table, individual comparison between non-IPF, CTD-ILD and Sarcoidosis patients with IPF patients did not yield statistically significant difference (p=0.39, 019 and 0.16 respectively).

S6 Table: Comparison of ILD specific pharmacotherapies of various ILDs w.r.t IPF

ILD specific pharmacotherapy	χ^2	p-Value
IPF vs non-IPF	0.75	0.39
IPF vs CTD-ILD	1.69	0.19
IPF vs Sarcoidosis	1.94	0.16

A comparison to compare compliance of ILD-specific pharmacotherapies between high volume (≥ 201 patients annually), moderate volume (100-200 patients annually) and low volume (≤ 100 patients annually) centres was also done. The volume was calculated based on our currently undergoing nation-wide ILD registry, ICMR's Network of Pulmonary Fibrosis (INPF). There was no significant difference between compliance of different volume categories (S7 Table).

S7 Table: Comparison of GCP and GNCP ILD-specific pharmacotherapies

Centre patient volume	GCP	GNCP	χ²	p-Value
Low (≤ 100 patients)	43 (74.1%)	15 (25.9%)	1.72	0.42
Moderate (100-200 patients)	60 (77.9%)	17 (22.21%)		
High (≥ 201 patients)	90 (82.6%)	19 (17.4%)		

With respect to non ILD-specific pharmacotherapy, PJP prophylaxis was given to 22 out of 101 eligible CTD-ILD patients, 8 out of 24 eligible sarcoidosis patients, and 16 out of 59 patients with HP. Calcium and Vitamin-D were prescribed to all 5 IPF eligible IPF patients, 92 out of 115 eligible CTD-ILD patients, 28 out of 32 eligible sarcoidosis patients, and 61 out of 69 eligible HP patients. Acid suppressants were given to 37 out of 59 eligible IPF patients, 107 out of 154 eligible CTD-ILD patients, 30 out of 35 eligible sarcoidosis patients and 53 out of 74 eligible HP patients. Cough suppressants were given to all eligible ILD patients. As shown in table below, comparison prescription of these non-ILD specific pharmacotherapies did not differ significantly among different ILD sub-types (S8 table).

Among non-pharmacological prescription, vaccination advice was provided to 13.2% of eligible patients overall, with no significant difference in prescription behaviour across ILD phenotypes (12.3% in CTD-ILD, 8.6% in sarcoidosis, 14.9% in HP and 16.9% in IPF; χ^2=1.63, p=0.65). Pulmonary rehabilitation advice was given to 7.1% of eligible patients overall, with no significant difference in prescription behaviour across ILD phenotypes (6.5% in CTD-ILD, 2.9% in sarcoidosis, 6.8% in HP and 8.5% in IPF; χ^2=1.15, p=0.76) (S8 table).

S8 Table: Comparison of non-ILD specific pharmacotherapies and non-pharmacological advice among various ILD sub-types

	IPF	CTD-ILD	Sarcoidosi	HP	χ²	p-value
**Non ILD-specific pharmacotherapy**						
PJP prophylaxis prescribed, n (%)	-	22 (21.8)	8 (33.3)	16 (27.1)	1.59	0.45
Ca–Vitamin D supplementation, n (%)	5 (100)	92 (80.0)	28 (87.5)	61 (88.4)	3.64	0.30
Acid suppressant prescribed, n (%)	37 (62.7)	107 (69.5)	30 (85.7)	53 (71.6)	5.74	0.13
**Non-pharmacological advice**						
Vaccination advice given, n (%)	10 (16.9)	19 (12.3)	3 (8.6)	11 (14.9)	1.63	0.65
Pulmonary rehabilitation advice given, n (%)	5 (8.5)	10 (6.5)	1 (2.9)	5 (6.8)	1.15	0.76

Comparison of the non ILD-specific pharmacotherapies (PJP prophylaxis, Ca–Vitamin D supplementation and Acid suppressant) among high (more than 200 ILD per year), moderate (101 to 200 per year) and low (100 or less per year) patient volume centres were also done. PJP provision varied from 17.3% in low-volume centres to 32.3% in moderate-volume centres and 26.8% in high-volume centres; however, this difference was not statistically significant (χ^2=3.33, df=2, p=0.19). Calcium–Vitamin D supplementation was provided to 80.6% of eligible patients in low-volume centres, 90.6% in moderate-volume centres, and 84.7% in high-volume centres. There was no statistically significant difference in supplementation across centre-volume categories (χ^2=2.62, p=0.269). Acid-suppressant therapy was provided to 93.8% of eligible patients in low-volume centres, compared with 83.3% in moderate-volume centres and 79.3% in high-volume centres. There was a statistically significant difference in acid-suppressant provision across centre-volume categories (χ^2=9.10, p=0.011).

Among non-pharmacological prescription, vaccination advice was given to 7% of eligible patients in low-volume centres, 4.67% in moderate-volume centres and 20.18% in high-volume centres; while pulmonary rehabilitation advice was given to 0%, 0.93% and 13.3% in low, moderate and high patient volume centres respectively. These results indicate that advice of non-pharmacological prescription differ significantly among centres.

S9 Table: Comparison of non-ILD specific pharmacotherapies and non-pharmacological advice among various low, moderate and high patient volume centres

	**Low volume (≤ 100 patients), n (%)**	**Moderate volume (100-200 patients), n (%)**	**High volume (≥ 201 patients), n (%)**	**χ^2**	**p-value**
**Non ILD-specific pharmacotherapy**					
PJP prophylaxis prescribed, n (%)	9 (17.3)	20 (32.3)	40 (26.8)	3.33	0.19
Ca–Vitamin D supplementation, n (%)	54 (80.6)	58 (90.6)	150 (84.7)	2.62	0.27
Acid suppressant prescribed, n (%)	75 (93.8)	60 (83.3)	153 (79.3)	9.1	**0.01**
**Non-pharmacological advice**					
Vaccination advice given, n (%)	7 (7.0)	5 (4.67)	44 (20.18)	19.45	**< 0.001**
Pulmonary rehabilitation advice given, n (%	0 (0)	1 (0.93)	29 (13.3)	26.67	**< 0.001**
