# Supplementary material for: Interstitial Lung Diseases treatment prescription practices among pulmonologists in India: A prospective multicentre observational study (India-ILD care study)
Source: PLoS One. 2026 Oct 23;21(10):e0360678. doi: 10.1371/journal.pone.0360678 (PMC13693893; doi:10.1371/journal.pone.0360678)
Supplement: Supplementary file 2 [file pone.0360678.s002.pdf]

SUPPLEMENTARY APPENDIX 2

Table: List of ICMR's Network of Pulmonary Fibrosis (INPF) collaborators

Sl. No	First & Middle Names	Surname	Affiliation/ Institute
1.	Randeep	Guleria	Department of Pulmonary, Critical Care and Sleep Medicine, AIIMS, New Delhi
2.	Deepali	Jain	Department of Pathology, AIIMS, New Delhi
3.	Sudheer	Arava	Department of Pathology, AIIMS, New Delhi
4.	Danveer	Bhadu	Department of Rheumatology, AIIMS, New Delhi
5.	Pritinanda	Mishra	Department of Pathology, AIIMS, Bhubaneswar
6.	Anupam	Dey	Department of General Medical, AIIMS, Bhubaneswar
7.	Sunitha Vellathussery	Chakkalakkoombil	Department of Radiodiagnosis JIPMER, Pondicherry
8.	Debasis	Gochhait	Department of Pathology, JIPMER, Pondicherry
9.	Molly Mary	Thabah	Department of Clinical Immunology, JIPMER, Pondicherry
10.	Chengappa G	Kavadichanda	Department of Clinical Immunology, JIPMER, Pondicherry
11.	Pankaj	Sharma	Department of Diagnostic and Interventional Radiology, AIIMS Rishikesh
12.	Venkatesh S	Pai	Department of Internal Medicine, AIIMS Rishikesh
13.	Sudipta	Pandit	Department of Clinical Immunology and Rheumatology IPGME&R and SSKM Hospital, Kolkata
14.	Somnath	Kundu	Department of Respiratory Medicine, IPGME&R, SSKM Hospital, Kolkata
15.	Keya	Basu	Professor and Head Department of Oncopathology, IPGME&R, SSKM Hospital
16.	Rumana	Makhdoomi	Department of Pathology, SKIMS, Soura, Srinagar
17.	Nasser Ahmad	Choh	Department of Radiology, SKIMS, Soura, Srinagar
18.	Farooq Ahmad	Ganie	Department of CVTS, SKIMS, Soura, Srinagar
19.	Ajai Kumar	Tentu	Department of Pulmonary Medicine, Bharati Vidyapeeth Medical College, Pune
20.	Narayanan	S	Department. of Pulmonary & Critical Care Medicine Army Hospital (R&R), Delhi
21.	Abhishek	Kumar	Department of Rheumatology), Army Hospital (R&R), Delhi
22.	Ashutosh Nath	Aggarwal	Department of Pulmonary Medicine, PGIMER, Chandigarh

23.	Ritesh	Agarwal	Department of Pulmonary Medicine, PGIMER, Chandigarh
24.	Kuruswamy Thurai	Prasad	Department of Pulmonary Medicine, PGIMER, Chandigarh
25.	Mandeep	Garg	Department of Radiodiagnosis and Imaging, PGIMER, Chandigarh
26.	Amanjit	Bal	Department of Histopathology, PGIMER, Chandigarh
27.	Zafar	Niyaz	Department of Radiodiagnosis, SGPGIMS, Lucknow
28.	Vinita	Agarwal	Department of Pathology, SGPGIMS, Lucknow
29.	Vikas	Agarwal	Department of Clinical Immunology, SGPGIMS, Lucknow
30.	Mansi	Gupta	Department of Pulmonary Medicine, SGPGIMS, Lucknow
31.	Nishant Kumar	Chauhan	Department of Pulmonary Medicine, AIIMS Jodhpur
32.	Gopal	Durgeshwar	Department of Pulmonary Medicine, AIIMS Jodhpur
33.	Hariharan	Iyer	Department of Pulmonary Medicine, AIIMS Jodhpur
34.	Nitesh	Gupta	Department of Pulmonary Medicine, VMMC and Safdarjung Hospital, New Delhi
35.	Rajnish	Kaushik	Department of Pulmonary Medicine, VMMC and Safdarjung Hospital, New Delhi
36.	Pranav	Ish	Department of Pulmonary Medicine, VMMC and Safdarjung Hospital, New Delhi
37.	Sufiyan	Zaheer	Department of Pathology VMMC and Safdarjung Hospital, New Delhi, India
38.	Swarna		Department of Radiology, VMMC and Safdarjung Hospital, New Delhi, India
39.	Ramya	Janardan	Department of Pulmonary Medicine, SJNAHS, Bengaluru
40.	Maria	Bukelo	Department of Pathology SJNAHS, Bengaluru
41.	Sankaralingam	Rajeswari	Department of Rheumatology, SRIHER, Chennai, Tamil Nadu
42.	Meena	Iyer	Department of Clinical Research, SRIHER, Chennai, Tamil Nadu
43.	Sheila	Elangovan	SRFAHS, Division of Radiology & Imaging Sciences, SRIHER, Chennai, Tamil Nadu
44.	Kiran	Gopinathan	Division of Radiology & Imaging Sciences, SRIHER, Chennai, Tamil Nadu
45.	Gramani Arumugam	Vasugi	Department of Pathology, SRIHER, Chennai, Tamil Nadu
46.	Gareth Y	Lyngwa	Department of Radiodiagnosis and Imaging, NEIGRIHMS, Shillong.
47.	Rupalika	Yadav	Department of Pulmonary, Critical Care and Sleep Medicine, AIIMS, New Delhi
48.	Jyoti	Yadav	Department of Pulmonary, Critical Care and Sleep Medicine, AIIMS, New Delhi

49.	Madhulika	Sharma	Department of Pulmonary, Critical Care and Sleep Medicine, AIIMS, New Delhi
50.	Shalini	Sharma	Department of Pulmonary Medicine & Critical Care, AIIMS, Bhubaneswar.
51.	Jyoti		Department of Respiratory Medicine, VPCI, New Delhi
52.	Arunmozy	E	Department of Pulmonary Medicine, JIPMER, Pondicherry
53.	Uma	Chaudhary	Department of Pulmonary Medicine, AIIMS, Rishikesh
54.	Sulagna	Chatterjee	Department of Pulmonary Medicine, IPGME&R, SSKM Kolkata
55.	Ishfaq Ahmad	Bhat	Department of Pulmonary Medicine, SKIMS, Soura, Srinagar
56.	Kiran	Jagtap	Department of Pulmonology, Critical Care and Sleep Medicine, AFMC, Pune
57.	Kulshreshth	Sinha	Department of Pulmonary, Critical Care and Sleep Medicine, AIIMS, Raipur.
58.	Jaya	Malik	Department of Respiratory Medicine, Army Hospital (R&R), Delhi
59.	Deeksha		Department of Pulmonary Medicine, PGIMER, Chandigarh
60.	R A	Pragati	Department of Pulmonary Medicine, SGPGIMS, Lucknow
61.	Rohit	Kumar	Department of Pulmonary Medicine, AIIMS Jodhpur
62.	Pawan	Rai	Department of Pulmonary Medicine, VMMC & Safdarjung Hospital, New Delhi
63.	Aiswarya		Department of Clinical Research, SRIHER, Chennai
64.	Mebalari	Lyngdoh	Department of TB and Respiratory Diseases, NEIGRIHMS Hospital, Shillong
